# The UGG Isoacceptor of tRNA^Pro^ Is Naturally Prone to Frameshifts

**DOI:** 10.3390/ijms160714866

**Published:** 2015-07-01

**Authors:** Howard B. Gamper, Isao Masuda, Milana Frenkel-Morgenstern, Ya-Ming Hou

**Affiliations:** 1 Department of Biochemistry and Molecular Biology, Thomas Jefferson University, Philadelphia, PA 19107, USA; E-Mails: howard.gamper@jefferson.edu (H.B.G.); isao.masuda@jefferson.edu (I.M.); 2 Faculty of Medicine, Bar-Ilan University, Henrietta Szold 8, Safed 1311502, Israel; E-Mail: milana.morgenstern@biu.ac.il

**Keywords:** +1-frameshift, +2-frameshift, cmo^5^U34, m^1^G37-tRNA, slippery mRNA sequence, ribosome, translation

## Abstract

Native tRNAs often contain post-transcriptional modifications to the wobble position to expand the capacity of reading the genetic code. Some of these modifications, due to the ability to confer imperfect codon-anticodon pairing at the wobble position, can induce a high propensity for tRNA to shift into alternative reading frames. An example is the native UGG isoacceptor of *E. coli* tRNA^Pro^ whose wobble nucleotide U34 is post-transcriptionally modified to cmo^5^U34 to read all four proline codons (5ʹ-CCA, 5ʹ-CCC, 5ʹ-CCG, and 5ʹ-CCU). Because the pairing of the modified anticodon to CCC codon is particularly weak relative to CCA and CCG codons, this tRNA can readily shift into both the +1 and +2-frame on the slippery mRNA sequence CCC-CG. We show that the shift to the +2-frame is more dominant, driven by the higher stability of the codon-anticodon pairing at the wobble position. Kinetic analysis suggests that both types of shifts can occur during stalling of the tRNA in a post-translocation complex or during translocation from the A to the P-site. Importantly, while the +1-frame post complex is active for peptidyl transfer, the +2-frame complex is a poor peptidyl donor. Together with our recent work, we draw a mechanistic distinction between +1 and +2-frameshifts, showing that while the +1-shifts are suppressed by the additional post-transcriptionally modified m^1^G37 nucleotide in the anticodon loop, the +2-shifts are suppressed by the ribosome, supporting a role of the ribosome in the overall quality control of reading-frame maintenance.

## 1. Introduction

Natural tRNAs in all three domains of life have evolved to contain an extensive array of post-transcriptional modifications to their nucleotide bases and backbones. Many of these modifications are targeted to the wobble nucleotide at position 34 and to the nucleotide at position 37 on the 3ʹ side of the anticodon. While modifications to both positions play an essential role in maintaining the protein synthesis reading frame, modifications to the wobble position have the unique role of expanding the capacity of tRNA to read the genetic code [1]. However, such an extension requires the modified wobble nucleotide to recognize multiple codons at the 3rd position, each through an imperfect base pair. This presents a conflict for the modification at the wobble position between the potential to maximize codon-anticodon pairing interactions and the propensity to induce the wobble base to shift into alternative frames due to imperfect base pairing interactions. How this conflict is resolved is poorly understood.

Frameshifts occur when a translating ribosome shifts into alternative reading frames in the forward or reverse direction. Some mRNA sequences are “programmed” with a frameshift event as a regulatory mechanism of gene expression, usually by engaging a one-nucleotide shift in either direction. For example, the *prfB* gene for the *E. coli* ribosome release factor 2 (RF2) contains an internal stop codon UGA. When intracellular levels of RF2 are high, termination of protein synthesis at the internal UGA codon dominates, producing a truncated and inactive protein that is rapidly degraded, whereas when RF2 levels are low, the internal UGA codon is by-passed via a forward +1-frameshift (+1FS) event, leading to the production of a full-length protein [2,3]. Such a +1FS event is genetically programmed and has a high efficiency, ranging from 30% to 100% depending on the cellular condition [4]. Another example is the backward −1-frameshift (−1FS) event that controls expression of the *E. coli* *dnaX* gene upon ribosomal translation at a slippery sequence followed by a downstream mRNA hairpin or pseudoknot structural barrier [5]. This programmed −1FS event reaches an efficiency of 80%, yielding a 4:1 product ratio between the γ subunit and the τ subunit of DNA polymerase III [6,7]. In contrast, non-programmed frameshift events are considered translational errors, arising from shifting of a tRNA-ribosome complex on slippery mRNA sequences made up of repetitive nucleotides. In the case of non-programmed +1FS events, the frequency is typically low, estimated to be less than one per 30,000 amino acids (or less than 0.003%) [8]. Unlike mis-sense errors, which replace one amino acid with another but permit the synthesis of full-length proteins, frameshift errors are more deleterious, because they change the reading frame and introduce a premature termination codon, with the potential to arrest cell growth.

**Figure 1:**
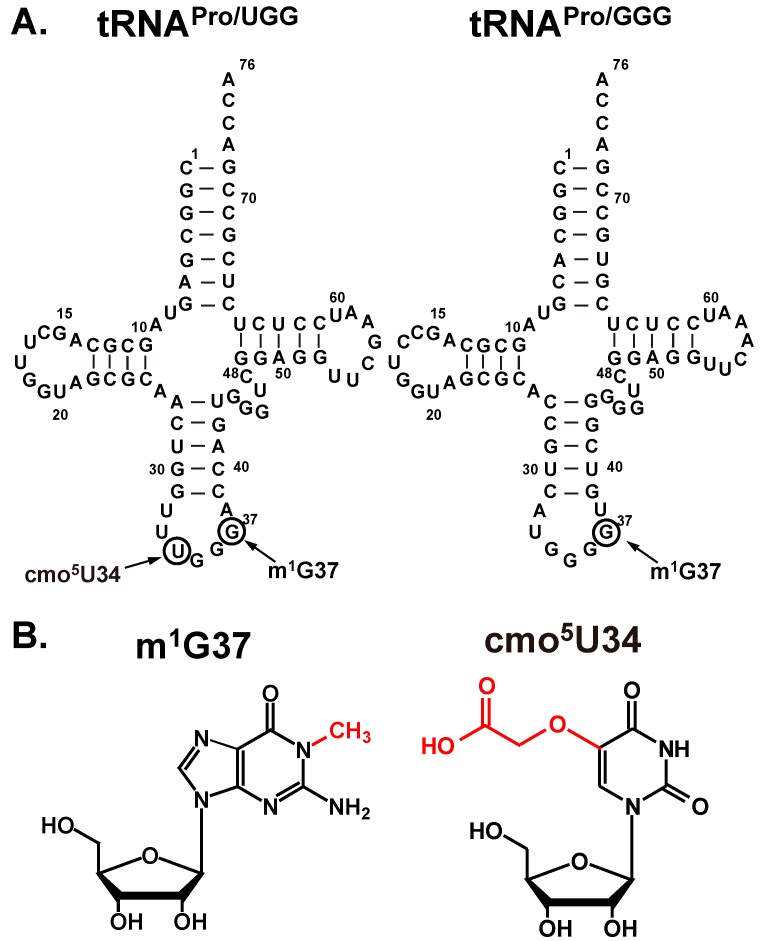
Sequence and cloverleaf structure of the UGG and GGG isoacceptors of *E. coli* tRNA^Pro^. (**A**) The sequence and nucleotide numbering is shown in the standard tRNA framework [19]. The nucleotides subject to post-transcriptional modifications from U34 to cmo^5^U34 and from G37 to m^1^G37 are indicated by circles; and (**B**) The chemical structure of cmo^5^U and m^1^G, showing the modification in red.

The mRNA sequences CC[C/U]-[C/U] are particularly slippery and are prone to inducing +1FS errors [9]. In the case of the CCC-C sequence, for example, the cognate tRNAs are the GGG and UGG isoacceptors of tRNA^Pro^. The GGG isoacceptor recognizes the codon sequence using three G-C base pairs, whereas the UGG isoacceptor relies on a post-transcriptional modification of U34 to cmo^5^U34 (5-oxyacetyl uridine) to pair with the codon (Figure 1) [10]. Both isoacceptors read the slippery sequence in the in-frame (0-frame) or the +1-frame with identical stability, indicating no energetic penalty for either tRNA to shift into the +1-frame. Indeed, we showed that when the CCC-C is placed at the 2nd codon position next to the AUG start codon in a reporter gene, it induces a frequency of +1FS errors as high as 1% in *E. coli* [11], significantly higher than the average frequency of non-programmed events. In both isoacceptors, a post-transcriptionally modified m^1^G37 nucleotide is conserved on the 3ʹ side of the anticodon (Figure 1) to maintain the reading frame. Removal of m^1^G37 from both isoacceptors increased the frequency of +1FS errors by 8-fold and brought *E. coli* cells to near death [11], supporting the notion that m^1^G37 is essential for cell survival [12,13,14]. Our kinetic studies further showed that +1FS errors of either tRNA can occur in one of two mechanisms: a slow shift from the P-site next to an empty A-site in a stalled post-translocation complex (post-complex) or a fast shift during translocation of tRNA into the P-site [11]. The slow shift is consistent with previous genetic studies [15,16,17,18] and is relevant during nutrient starvation that depletes cellular supplies of aminoacyl-tRNAs to the A-site. In contrast, the fast shift is relevant during active cell growth, because it occurs at a rate comparable to the normal rate of peptide bond formation [11].

Between the two isoacceptors, the UGG isoacceptor is of high interest. The cmo^5^U34 wobble base enables this tRNA to read all four Pro codons (5ʹ-CCN), making the tRNA the major isoacceptor for Pro and essential for cell survival [10]. However, the cmo^5^U34 also subjects the tRNA to a high propensity of frameshifting at the CC[C/U]-N sequence motif (N = A, C, G, U), because of the expanded pairing capacity of the modified wobble base. Thus, the native-state of the UGG isoacceptor presents a conundrum: while the cmo^5^U34 wobble base is important for maintaining cellular life, it also predisposes the tRNA to frameshifting. Among *E. coli* protein coding genes, the sequence motif CC[C/U]-N occurs 12,874 times, whereas the sequence motif CC[C/U]-[C/U] occurs only 2353 times. Of the 12,874 occurrences, a notable fraction of the CC[C/U] codons are followed by a rare codon (12.1%, Table 1), which can further increase the propensity of forward frameshifting [11,20]. Therefore, because the UGG isoacceptor poses a major challenge to reading-frame maintenance relative to its GGG counterpart, a better understanding of how the UGG isoacceptor translates slippery mRNA sequences is warranted.

Here we report that the native-state UGG isoacceptor is inherently shift-prone. Using the slippery CCC-CG mRNA sequence as an example, we show that this tRNA can preferentially shift not by one base but by two bases, resulting in a +2-frameshift (+2FS) error. The propensity for the +2FS event is driven by the more stable codon-anticodon base pairing of the wobble position relative to the 0-frame or +1-frame. Importantly, despite the high propensity, the +2FS event does not support subsequent peptide synthesis, leading to immediate termination of protein synthesis at the site of the shift. This is a major distinction from the +1FS event, which we have shown supports subsequent rounds of peptide synthesis and allows the ribosome to continue up to a premature stop codon [11]. The involvement of the ribosome in arresting protein synthesis upon a +2FS event emphasizes the contribution of the ribosome to maintaining the reading frame [21]. This study, together with our recent study of the UGG isoacceptor [11], provides new insight into how cells resolve the dilemma of having the cmo^5^U34 modification in the tRNA. While the modification induces both +1FS and +2FS errors, they are suppressed differently; +1FS errors are suppressed by the m^1^G37 modification on the tRNA, whereas +2FS errors are suppressed by the ribosome. We suggest that the ribosome does not recognize +1FS events as errors, because such events were developed in programmed shifts as a regulatory mechanism of gene expression.

## 2. Results

### 2.1. The Native-State UGG tRNA^Pro^ Is Unable to Fully Convert a Dipeptide to a Tripeptide

**Figure 2:**
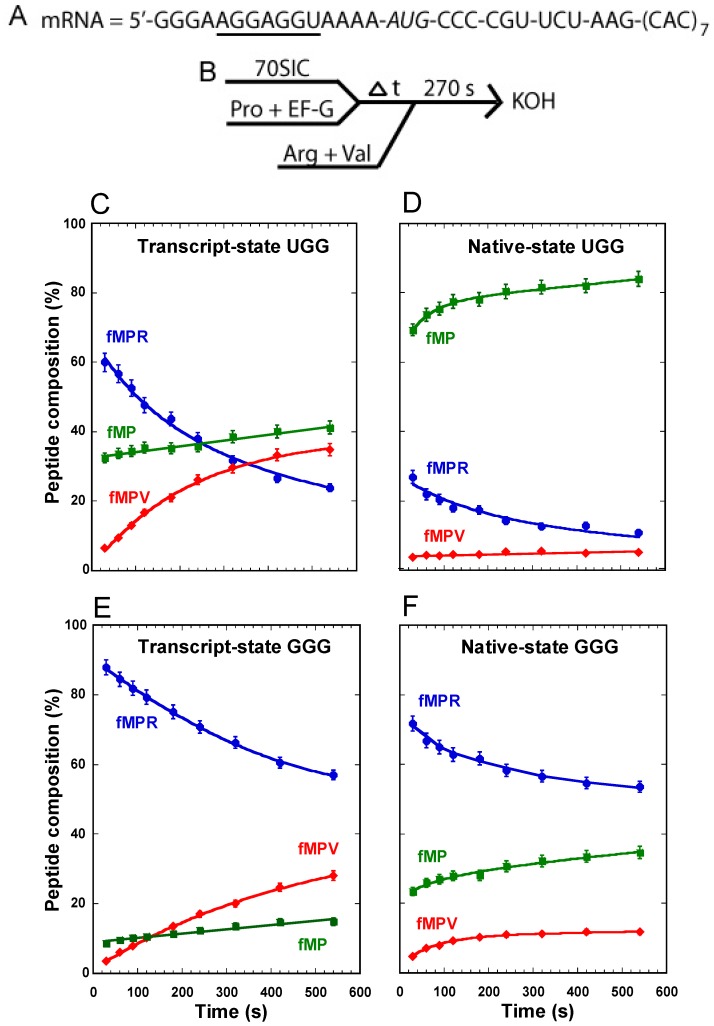
Analysis of forward +1 frameshifts of tRNA^Pro^ from a stalled ribosome complex. (**A**) The template mRNA sequence, showing the 5ʹ-Shine Dalgarno sequence (underlined), the AUG start codon (italic), and the slippery CCC-C sequence immediately following the start codon; (**B**) The reaction scheme for the conversion from fMP to tripeptides, starting with mixing a 70SIC with the ternary complex of tRNA^Pro^ in the presence of EF-G to form a post-complex with fMP-tRNA^Pro^ in the P-site. After stalling for various time periods, the complex was reacted with ternary complexes of tRNA^Arg^ and tRNA^Val^ for 270 s before being quenched by KOH. The changes in the peptide composition over time for the substrate fMP, the 0-frame product fMPR, and the +1-frame fMPV were monitored for (**C**) the transcript-state of the UGG isoacceptor of tRNA^Pro^; (**D**) the native-state of the UGG isoacceptor; (**E**) the transcript-state of the GGG isoacceptor; and (**F**) the native-state of the GGG isoacceptor. Each data point is the average of 3 independent measurements.

We have used the mRNA sequence 5ʹ-AUG-CCC-CGU-U to study non-programmed +1FS events (Figure 2A) [11]. In this sequence, the slippery CCC-C motif is next to the AUG start codon, which is the most shift-prone position in the entire reading frame [11]. For a translating *E. coli* ribosome carrying the fMP dipeptide on tRNAPro (fM = formyl methionine) in the post-complex, the correct reading of the slippery motif followed by one more round of peptide bond formation will generate the tripeptide fMPR, whereas the +1-frame reading will generate the tripeptide fMPV. These alternative products are readily resolved by electrophoretic thin layer chromatography (eTLC), allowing quantitative analysis of the fractional conversion from the dipeptide fMP to the tripeptide fMPR or fMPV at the site of the CCC-C motif. Using this mRNA sequence, we developed an assay to detect tRNA^Pro^ shifting into the +1-frame in a post-complex [11]. Specifically, we assembled the post-complex by taking the *E. coli* 70S initiation complex (70SIC) through the first round of peptide bond formation to place fMP-tRNA^Pro^ in the P-site next to an empty A-site (Figure 2B). By allowing fMP-tRNA^Pro^ to shift into the +1-frame over time, we quenched the shift by simultaneously adding ternary complexes of tRNA^Arg^ and tRNA^Val^ to take the ribosome through the next peptide bond formation. It was with this assay that we observed the synthesis of fMPV tripeptide, indicating that tRNA^Pro^ can shift into the +1-frame [11]. Control experiments confirmed that the synthesis of fMPV indeed reflected the +1-frame shift of tRNA^Pro^, rather than the miscoding by tRNA^Val^ [11]. However, the shift from a stalled post-complex occurs at a slow rate relative to the rate of peptide bond formation [11], suggesting that it is relevant only when the A-site is empty or contains a rare codon.

Our recent study with this assay primarily focused on the synthesis of fMPV [11] without much attention to the unreacted fMP substrate or the 0-frame product fMPR. To gain more insight into all three components and their quantitative changes over time in the course of the reaction, we applied this assay to study the UGG isoacceptor of tRNA^Pro^ and analyzed the fractional composition of each peptide among all three. With the transcript-state of the tRNA, made by *in vitro* transcription and lacking all natural post-transcriptional modifications, we observed a progressive accumulation of the fMPV product from background to 40%, a rapid synthesis of the fMPR product to 60% followed by a decrease to 20%, and a substantial consumption of the substrate fMP down to 40%, which remained relatively stable over the course of the 10-min reaction (Figure 2C). The stable level of the remaining fMP dipeptide, together with the steady increase of the fMPV tripeptide and the concomitant decrease of the fMPR tripeptide, indicates that the substrate was readily consumed, as the tRNA gradually shifted from the 0-frame into the +1-frame for the next round of peptide bond formation.

With the native-state of the UGG tRNA, isolated from cells and containing all natural post-transcriptional modifications, we observed a suppression of the synthesis of the fMPV product to only ~2%, a suppression of the synthesis of the fMPR product to 10%, but surprisingly little consumption of the substrate fMP, which remained at 80% (Figure 2D). Relative to the transcript-state, the suppression of the synthesis of fMPV in the native-state is attributable to the presence of m^1^G37, but not cmo^5^U34. In fact, a variant of the native-state, containing cmo^5^U34 but lacking m^1^G37, was as shift-prone as the transcript-state, showing high activity of synthesis of the fMPV product [11]. This indicates that it is m^1^G37 that is the major determinant for suppressing +1FS errors of this tRNA. However, the high level of unconsumed fMP in the native-state was unexpected.

For comparison, the transcript-state of the GGG isoacceptor behaved similarly as the UGG counterpart, enabling a steady increase of the synthesis of the fMPV product, a steady decrease of the fMPR product, and a substantial consumption of the substrate fMP down to 20% (Figure 2E). In the native-state, we also observed a suppression of the synthesis of fMPV, attributable to the presence of m^1^G37 (Figure 2F) [11]. While the suppression of the synthesis of fMPV was not as effective as in the UGG isoacceptor, this is because the reaction lacked the protein elongation factor EF-P, which is required to completely suppress +1FS errors of the GGG isoacceptor [11]. However, a major difference was observed for the substrate fMP; while it was consumed to below 40% when attached to the native-state GGG isoacceptor, it was hardly consumed when attached to the native-state UGG isoacceptor (Figure 2D *vs.* F).

**Figure 3:**
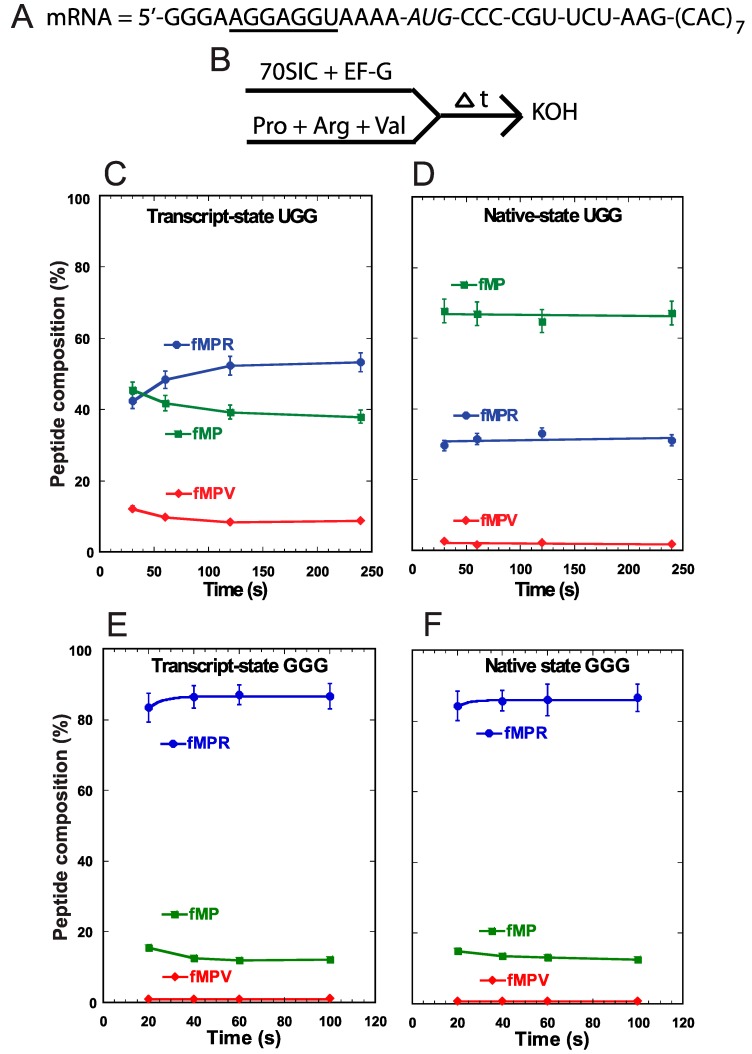
Analysis of forward +1 frameshifts of tRNA^Pro^ during translocation into the P-site. (**A**) The template mRNA sequence, showing the 5ʹ-Shine Dalgarno sequence (underlined), the AUG start codon (italic), and the slippery CCC-C sequence immediately following the start codon; (**B**) The reaction scheme for the conversion from fMP to tripeptides, starting with rapid mixing of a 70SIC with ternary complexes of tRNA^Pro^, tRNA^Arg^, and tRNA^Val^, in the presence of EF-G. The reaction was quenched over time by KOH. The changes in the peptide composition over time for the substrate fMP, the 0-frame product fMPR, and the +1-frame fMPV were monitored for (**C**) the transcript-state of the UGG isoacceptor of tRNA^Pro^; (**D**) the native-state of the UGG isoacceptor; (**E**) the transcript-state of the GGG isoacceptor; and (**F**) the native-state of the GGG isoacceptor. Each data point is the average of 3 independent measurements.

We performed a different assay to monitor +1FS errors during translocation of tRNA^Pro^ into the P-site. In this assay, we assembled the 70SIC on the same slippery mRNA sequence (Figure 3A) and took the ribosome complex through two successive rounds of peptide bond formation in the presence of EF-G and ternary complexes of tRNA^Pro^, tRNA^Arg^, and tRNA^Val^ (Figure 3B). Because the A-site was occupied throughout the assay, and because there is no evidence of tRNA shifting in the A-site [11], the synthesis of fMPV indicated that tRNA^Pro^ had shifted to the +1-frame during its translocation into the P-site. We showed recently that the rate of +1-shifting during translocation was fast [11], comparable to the rate of normal peptide bond formation, suggesting its potential to challenge the reading-frame maintenance in actively growing cells. Using this concerted assay, we detected low levels of accumulation of the +1-frame product fMPV with the UGG isoacceptor in both the transcript-state and the native-state (Figure 3C,D). In fact, because the level of fMPV did not change appreciably between the two states, this supports the notion that +1FS errors during tRNA translocation are not suppressed by m^1^G37 or other post-transcriptional modifications [11]. Importantly, while both states also synthesized the 0-frame product fMPR to similar levels, they differed in the consumption of the fMP substrate. While the transcript-state showed a time-dependent consumption of fMP down to 40%, the native-state maintained a high level of fMP (>60%) with little decrease over time (Figure 3C,D). The inability to convert fMP to either product was unique to the native-state of the UGG isoacceptor and was not observed with the GGG isoacceptor. For the latter, the fMP substrate was readily converted to the 0-frame product fMPR at high levels and to the +1-frame product fMPV at low levels, independent of the transcript- or native-state (Figure 3E,F). These results confirmed that +1FS errors are typically low during tRNA translocation into the P-site and that such errors are not suppressible by post-transcriptional modifications. Importantly, unlike the UGG isoacceptor, the GGG isoacceptor in both the transcript- and native-state consumes the fMP substrate to below 20%.

### 2.2. Base Pairing Stability of cmo^5^U34 in the Native-State of the UGG Isoacceptor

**Figure 4:**
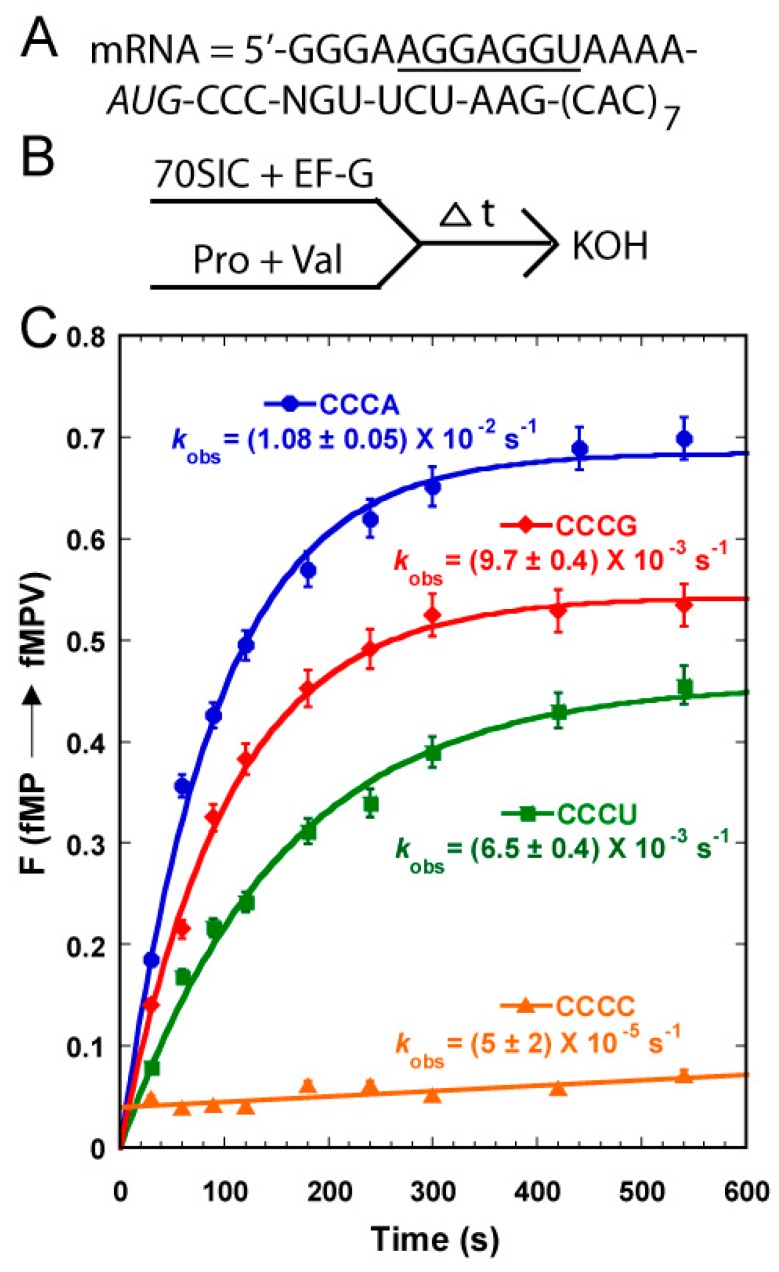
The propensity of shifting into the +1-frame of the native-state UGG isoacceptor of tRNA^Pro^ on mRNA CCC-N sequences. (**A**) The template mRNA sequences, showing the 5ʹ-Shine Dalgarno sequence (underlined), the AUG start codon (italic), and the slippery CCC-N sequence immediately following the start codon, where N = A, C, G, and U; (**B**) The reaction scheme for monitoring +1FS by rapid mixing of a 70SIC with ternary complexes of tRNA^Pro^ and tRNA^Val^ in the presence of EF-G; and (**C**) Kinetics of the fractional conversion (F) from fMP to fMPV was monitored over time. The *k*_obs_ for each mRNA sequence was indicated and each data point is the average of 3 independent measurements.

The unusually high level of unreacted fMP was observed only with the native-state of the UGG isoacceptor, which set this tRNA apart from the other three studied above. One possibility was that this tRNA preferentially dropped off from the ribosome relative to the other three. This was unlikely, given that it is the major form of tRNA^Pro^ and that it is in the fully modified state, which is more robust in the binding and activity with the ribosome than the transcript-state [11]. We thus considered the possibility that, due to the possession of cmo^5^U34, the native-state UGG isoacceptor is highly shift-prone on a slippery mRNA sequence and can shift into alternative frames other than the +1-frame. This possibility is based on the unusual pairing stability of cmo^5^U34. While the cmo^5^U34-containing UGG isoacceptor can recognize all four 5ʹ-CCN codons for Pro, stability of the wobble base pair in the codon-anticodon duplex varies greatly among the four. Crystal structural analysis of ribosome-tRNA complexes suggested that the pairing of cmo^5^U34 with A or G is the most stable, followed by the pairing with U, and by the pairing with C [11]. To test this hypothesis, we took 70SIC through two rounds of peptide bond formation with ternary complexes of the native-state UGG isoacceptor and the +1-frame tRNA^Val^ (Figure 4A,B). In this design, the post-complex of fMP-tRNA^Pro^ after the first peptide bond formation must shift into the +1-frame to react with tRNA^Val^ to synthesize the fMPV product. The speed and level of fMPV synthesis would be driven by the stability of the pairing between its cmo^5^U34 and the N nucleotide on the mRNA. A time-dependent analysis showed that the fractional conversion was the fastest and reached the highest level with the CCC-A mRNA sequence, followed by the CCC-G mRNA, and by the CCC-U mRNA, with the conversion being the poorest with the CCC-C mRNA (Figure 4C). This relative shifting propensity, in both the kinetics and the overall level, was in complete agreement with the structural prediction, supporting the notion that base pairing stability of cmo^5^U34-N at the wobble position is the major determinant to drive the shift. In contrast, the transcript-state of the same tRNA shifted with similar efficiency among the four mRNA sequences without a preference [11], suggesting that the pairing between the unmodified U34 and the N base lacked a thermodynamic selectivity. This comparison highlights the ability of the modified cmo^5^U34 relative to the unmodified U34 to differentiate among base pairing partners.

### 2.3. Evidence of the Native-State UGG tRNA^Pro^ Shifting into the +2-Frame

**Figure 5:**
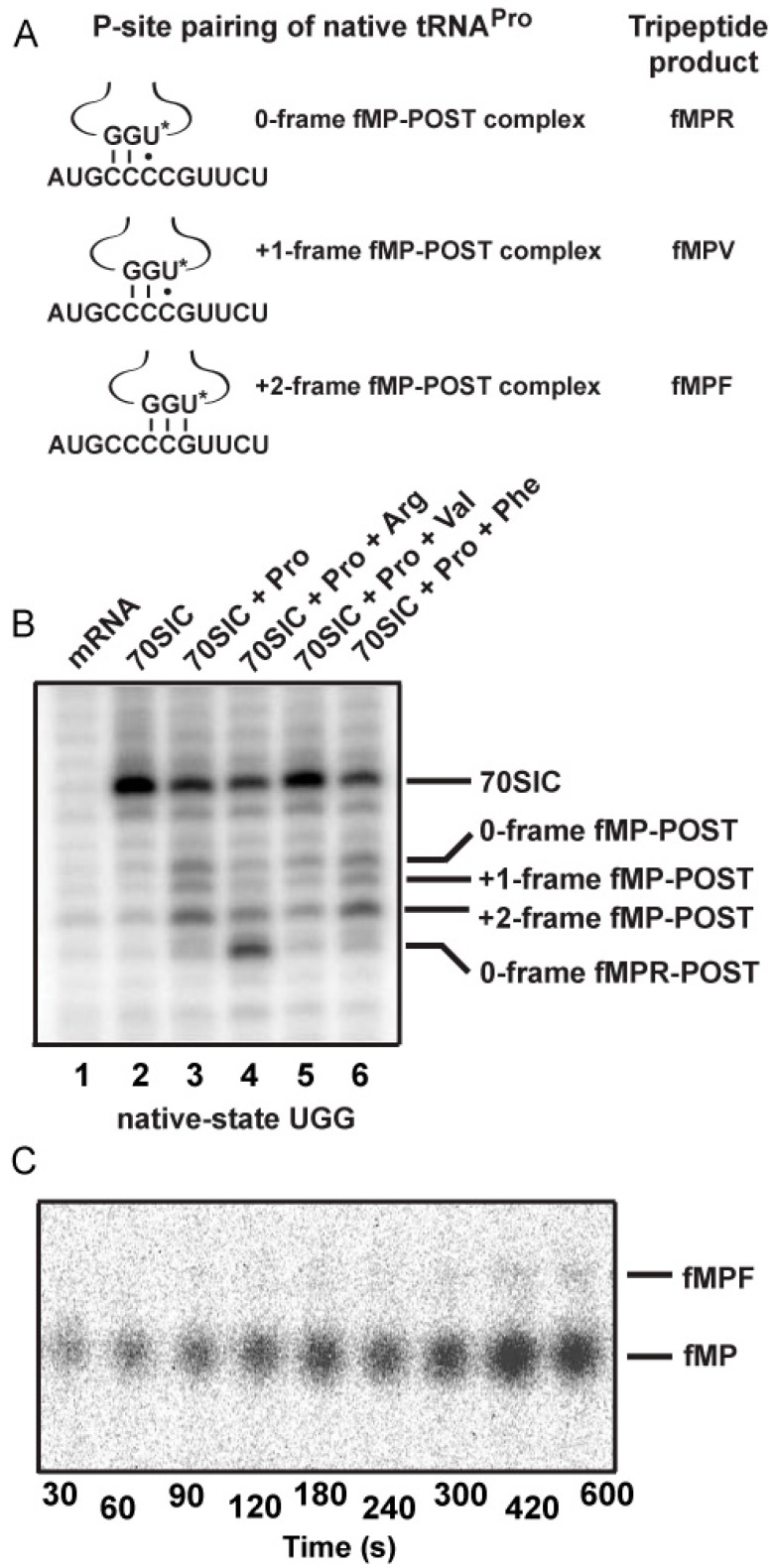
Dipeptidyl and tripeptidyl post-complexes formed on a slippery mRNA sequence. (**A**) Post-complexes were formed on a long mRNA that contained the initial AUG-CCC-CGU-UCU coding sequence as used in Figure 2 and Figure 3. The fMP-post complex is shown with the native-state UGG isoacceptor in the 0-, +1-, and +2-frame. The codon-anticodon pairing interaction and the tripeptide product of each after one more round of peptide bond formation is illustrated. The U* denotes cmo^5^U34; (**B**) Toeprints of ribosome post-complexes formed by interaction of 70SIC with ternary complexes of the indicated tRNAs in the presence of EF-G. Unbound mRNA was included as a control and the 70SIC with a P-site bound fMet-tRNA^fMet^ was used as a reference for the reading frame; and (**C**) eTLC analysis of +2-frame fMPF formation. 70SIC with an mRNA with the initial coding sequence of AUG-CCC-CGU-UCU was mixed with ternary complexes of native tRNA^Pro/UGG^ and tRNA^Phe/GAA^ in the presence of EF-G and incubated at 20 °C. Aliquots were quenched in 0.5 M KOH prior to analysis by eTLC.

With the native-state UGG isoacceptor, we tested if the high levels of unreacted fMP substrate (Figure 2C) was due to its shifting beyond the +1-frame to a position unable to participate in peptide bond formation. Based on the established order of relative stability, the reading of the cmo^5^UGG anticodon in the 0-frame or the +1-frame of a post-complex would be in the least stable context, involving the cmo^5^U-C base pair, whereas the reading in the +2-frame would be in a more stable context, involving the cmo^5^U-G base pair (Figure 5A). We hypothesized that the difference in stability would provide a strong thermodynamic drive in favor of the tRNA to shift, not only by one, but more preferably by two nucleotides into the +2-frame. In contrast, we hypothesized that the transcript-state containing the unmodified U34, which would pair with G poorly, lacked the thermodynamic drive to shift into the +2-frame. The poor pairing of an unmodified U34 with G is notable and has been reported previously [11].

Toeprint analysis supported our hypothesis, showing the shift into the +2-frame of the native-state UGG isoacceptor (Figure 5B). Using the same slippery mRNA sequence in the context of a longer mRNA, we formed post-complexes at various positions on the mRNA by mixing 70SIC with the ternary complex of the native-state UGG isoacceptor alone or in the presence of ternary complexes of the next 0-frame, +1-frame, or +2-frame tRNA. We then determined the position of the P-site in each complex by primer extension of an oligonucleotide annealed to the 3ʹ-end. In this design, the mRNA alone posed no block to primer extension (lane 1), while the 70SIC with fM-tRNA^fMet^ posed a block at the P-site (lane 2). The post-complex with each addition of an amino acid would move the P-site down by one codon distance toward the 3ʹ-end, thereby shortening the length of the primer extension accordingly. In the post-complex formed with 70SIC and the ternary complex of the native-state UGG tRNA^Pro^, the synthesized fMP dipeptide was found at all three frames but most prominently in the +2-frame (43% of all possible frames, lane 3), indicating that the tRNA had shifted from the 0-frame, transiently passed the +1-frame, and stably ended up at the +2-frame. In the post-complex formed with ternary complexes of tRNA^Pro^ and tRNA^Arg^ (the next 0-frame tRNA), the synthesized tripeptide fMPR was primarily found 6-nucleotide down from the 70SIC (58% of all possible frames, lane 4), indicating the movement of the tRNA-ribosome complex by two in-frame codons. The in-frame movement of the fMPR-post complex served as a positive control for tripeptide formation, although a notable fraction of the post-complex was also found 5-nucleotide down (lane 4), indicating a shift into the +2-frame before settling at the 6-nucleotide down position. However, in the post-complex formed with ternary complexes of tRNA^Pro^ and tRNA^Val^, primer extension stopped with the fMP dipeptide at the 0- or the +2-frame, but no stop with the fMPV tripeptide at the +1-frame (lane 5). This result is consistent with the notion that, once the fMP dipeptide was formed, a fraction of the native-state UGG tRNA stayed in the 0-frame and another fraction rapidly moved into the +2-frame, while the +1-frame fMPV tripeptide was barely detectable (Figure 5C), due to the low yield of production (~5%) as shown in kinetic analysis (Figure 4, mRNA = CCC-C).

Analysis of the post-complex formed with ternary complexes of tRNA^Pro^ and tRNA^Phe^ revealed an intriguing result. While tRNA^Phe^ was cognate to the UUC codon in the +2-frame (Figure 5A), primer extension was blocked with the fMP dipeptide primarily at the +2-frame (46% of all frames, lane 6) without evidence for the formation of a tripeptidyl-tRNA. This result suggests that the +2-frame ribosome complex failed to support peptide synthesis even with the cognate tRNA^Phe^. Indeed, eTLC analysis of reactions containing 70SIC and ternary complexes of the UGG isoacceptor and tRNA^Phe^ yielded a low yield of tripeptide (less than 1% of the substrate, Figure 5C), supporting the notion that the +2-frame post-complex was a poor substrate for peptide bond formation. Thus, because of the inability of the +2-frame post-complex to catalyze peptide bond formation, the data explain why the fMP substrate was not consumed with the native-state UGG tRNA^Pro^. In cellular conditions, such a +2-frame post-complex, stalled at the P-site and unable to perform peptidyl transfer, is likely resolved by the drop-off of the P-site tRNA or by the several rescue mechanisms available to bacterial ribosomes [22,23,24].

## 3. Discussion

This study revealed a high propensity of the native-state UGG tRNA^Pro^ to frameshift into the +2-frame, particularly at the slippery mRNA sequence CCC-CG placed at the 2nd codon position. The 2nd codon position is unique relative to others for the high ability to induce tRNA to frameshift both in the fast and slow mechanisms [11]. Several possibilities are envisioned. One is the absence of an E-site tRNA, which can promote reading-frame errors [25,26]; the second is the association of the ribosome complex with the Shine Dalgarno (SD) sequence, which can hamper the first translocation and promote tRNA shifting [27]; and the third is the involvement of the structurally distinct initiator tRNA^fMet^ that is unfavorable for translocation [28]. Of these three, the association of the ribosome complex with the SD sequence is of interest, because it allows the SD to form stable base pairs with the 3ʹ-end of the ribosomal 16S rRNA, thus causing the ribosome complex to stall and inducing tRNA to shift. The SD-16S rRNA interaction is most favorable when the distance between the 3ʹ-end of SD and the shift sequence is 7–10 nucleotides, which has promoted both −1FS in the *dnaX* sequence and +1FS in the *prfB* sequence [29]. The SD sequence and the CCC-C sequence in all of our assays is separated by 7 nucleotides, suggesting the possibility that the SD-16S rRNA interaction can be a component of the +2FS mechanism observed here.

In nature, non-programmed +2FS events are rare. One example was reported previously on an mRNA consisting of repetitive GA repeats, which are predisposed to slippage [30]. The cognate tRNA^Glu^ at the GA repeats normally contained the modified mnm^5^s^2^U34 at the wobble position. The +2FS event was observed by genetic analysis in *E. coli* cells lacking the synthesis of mnm^5^s^2^U34 and lacking sufficient quantities of the aminoacyl-tRNA for the A-site codon next to the Glu codon. It was suggested that when *E. coli* cells were deficient in MnmE and GidA, the two enzymes required for the synthesis of mnm^5^s^2^U34, the hypo-modified tRNA^Glu^ was prone to shift from the P-site in a post-complex. This propensity was further exacerbated by the insufficient occupancy of the A-site by the UCU isoacceptor of tRNA^Arg^. The choice of the shift to the +2-frame was rationalized, because the codon-anticodon pairing in the +2-frame would have the same stability as in the 0-frame, whereas a shift to the +1-frame would have less stability. This supports our notion that the thermodynamic stability of the codon-anticodon pairing of the wobble position is a driving force for determining the position of the shift. Although the study of tRNA^Glu^ suggested the synthesis of the +2FS protein product, based on the expression of a reporter *lacZ* gene on an indicator plate, no direct biochemical evidence for the protein product was available [30]. Possibly, even an extremely low level of the +2FS product would be sufficient to give rise to a positive response on an indicator plate, whereas it would be too low to be detected in a biochemical assay.

**Figure 6:**
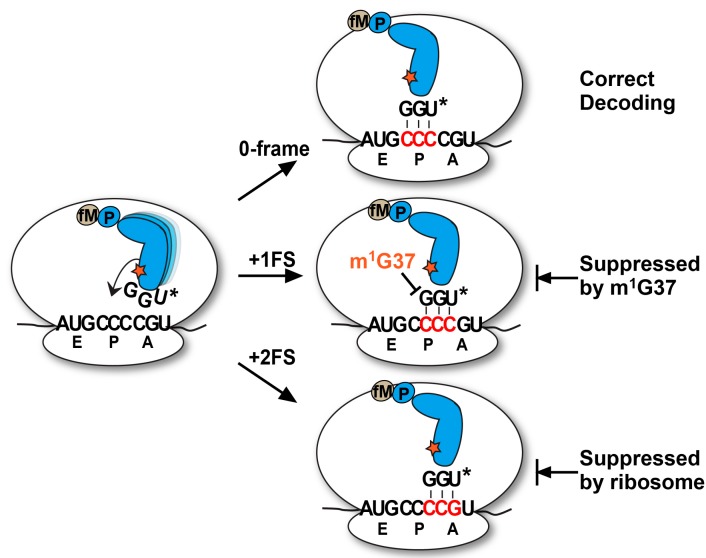
A model of the propensity of the UGG isoacceptor tRNA^Pro^ to shift due to the presence of the cmo^5^U34 base (shown as U*). The 0-frame decoding is shown on the top; the +1FS is shown in the middle, which is suppressed by m^1^G37 in the anticodon loop of the tRNA; and the +2FS is shown at the bottom, which is suppressed by the ribosome due to the lack of further peptidyl transfer. The tRNA is shown in blue, carrying the fMP dipeptide, and m^1^G37 is shown as a red star.

Another +2FS event was reported for a leaky −1FS allele *trpE91* in *Salmonella* [31]. The low level of translational frameshifting that occurred at this site was studied by transferring the region surrounding the mutation into a *lacZ* expression vector [32]. The sequence of the expressed protein product revealed a +2FS event [32]. Further analysis showed that the +2FS event was mediated by translational hopping, due to the UAC isoacceptor tRNA^Val^ leaving the ribosome complex and landing 2 nucleotides later on the mRNA GUGUA sequence, which has overlapping Val codons [33]. Interestingly, the UAC isoacceptor tRNA^Val^ also contains the modified cmo^5^U34 at the wobble position, although the hopping mechanism is distinct from the shifting mechanism of the UGG isoacceptor tRNA^Pro^ we report here.

Our analysis of the native-state UGG isoacceptor of tRNA^Pro^ supports a +2FS model on a slippery mRNA sequence. In this model, the pairing strength of the cmo^5^U34 wobble base with the third base of a codon provides a driving force for the tRNA to shift from the 0-frame, to transiently pass through the +1-frame, and to stably arrive at the +2-frame. The transient passage through the +1-frame is due to the similar pairing stability relative to the 0-frame, while the stable arrival at the +2-frame is due to the significantly improved pairing stability. Toeprint analysis shows that, once the tRNA reaches the P-site in the +2-frame, it does not slip back to the 0 or +1-frame, further supporting the thermodynamic prediction. Our kinetic analysis reveals that +2-frameshifts can occur both during and after tRNA translocation into the P-site, similar to the two mechanisms we described for +1-frameshifts [11]. However, we suggest that the cellular solution to suppress +1FS and +2FS errors is different (Figure 6). While +1FS errors are suppressed by the modified m^1^G37 conserved in the anticodon loop of tRNA^Pro^, these errors are not recognized by the ribosome, perhaps because ribosomes have evolved to accept +1FS events at programmed sites as a regulatory mechanism for gene expression. In contrast, +2FS errors are recognized by the ribosome and peptidyl transfer is arrested upon the shift, preventing further propagation of errors. Thus, besides post-transcriptional modifications in tRNA, the ribosome is an active component in the maintenance of the protein synthesis reading frame. While previous genetic studies had implicated a role of the ribosome in preventing +1FS errors [26], our work here establishes a role of the ribosome in preventing +2FS errors.

## 4. Experimental Section

### 4.1. Materials

This study used several native-state *E. coli* tRNAs, each of which was expressed from the pKK223-3 plasmid and purified from total tRNA by affinity hybridization to a biotin-tagged complementary oligonucleotide [34,35]. These included UGG and GGG isoacceptors of tRNA^Pro^, CAU isoacceptor of tRNA^fMet^, ICG isoacceptor of tRNA^Arg^ (I = inosine), U*AC isoacceptor of tRNA^Val^ (U* = cmo^5^U34), and GAA isoacceptor of tRNA^Phe^. Unmodified transcript-state tRNAs were prepared by *in vitro* transcription and purified by denaturing electrophoresis through a 12% PAGE/7 M urea gel [36,37]. Each tRNA was charged by its respective aminoacyl-tRNA synthetase and stored in the buffer 25 mM NaOAc pH 5. Formylation of Met-tRNA^fMet^ was carried out in the charging reaction by including methionyl formyltransferase in the presence of 10-formyltetrahydrofolate [35,38,39]. mRNAs were prepared by transcription off double-stranded DNA templates with T7 RNA polymerase [35]. The 70S ribosomes, initiation factors, and elongation factors were isolated from *E. coli* using standard procedures [38].

### 4.2. In Vitro Translation Assays

*In vitro* translation reactions were conducted in 50 mM Tris-HCl pH 7.5, 70 mM NH_4_Cl, 30 mM KCl, 3.5 mM MgCl_2_, 1 mM DTT, 0.5 mM spermidine, and 1–2 mM GTP [35,38,39]. In each reaction, the 70S ribosome was incubated with a limiting amount of ^35^S-fMet-tRNA^fMet^, excess initiation factors 1, 2 and 3, and mRNA for 25 min at 37 °C. Ternary complexes were formed by incubating a limiting amount of charged tRNA with GTP-activated EF-Tu for 15 min in an ice bath [40]. Translation was initiated by mixing the 70SIC with one or more ternary complexes in the presence of EF-G at 20 °C. The limiting reagent in these reactions was the initiator tRNA, which was present at 0.25 μM. Reaction aliquots were quenched by adding KOH to the final concentration of 0.5 M. After a brief incubation at 37 °C, aliquots of 1 μL or less were loaded onto TLC cellulose sheets for electrophoresis in PYRAC buffer (62 mM pyridine, 3.48 M acetic acid pH 2.7) [41]. Peptides were quantified by phosphorimaging as described [11].

### 4.3. Toeprint Analysis

Toeprinting of ribosomal complexes was performed as described [11]. Briefly, initiation or post complexes were formed on a 96-mer mRNA with an initial coding sequence of AUG-CCC-CGU-UCU-. Following complex formation, a 5ʹ-32P-primer that had previously been hybridized to the mRNA was extended to the edge of the ribosome using Superscript reverse transcriptase III (Life Technologies, Grand Island, NY, USA). Reactions were phenol extracted and ethanol precipitated. The products of primer extension were separated from the primer on a 40 cm long 12% PAGE/7 M urea gel and visualized by phosphor imaging. The position of each primer extension product was determined relative to a control sample containing fMet-tRNA^fMet^ in the P-site of the 70SIC.

**Table 1:** The number of CC[C/U] codons followed by a rare codon in the *E. coli* K12 protein-coding genes.

**Codon**	**CGA**	**CCC**	**CCA**	**CCU**	**CGG**	**CUA**	**UCA**	**UCG**	**UGC**	**UGU**	**Total**
**Codon Freq**	0.3	0.4	0.8	0.7	0.5	0.3	0.7	0.8	0.6	0.4	5.5
**CCC**	18	25	16	32	30	9	55	46	72	51	354
**CCU**	10	43	47	40	12	22	140	114	72	45	545
**Total**	28	68	63	72	42	31	195	160	144	96	899
**Codon**	**ACA**	**AGA**	**AGG**	**AGU**	**AUA**	**GGA**	**GGG**	**Total**	**% of Total Number of CC[C/U]-[NNN] (12,874)**		
**Codon Freq**	0.1	0.2	0.2	0.7	0.4	0.7	0.9	3.2			
**CCC**	59	30	13	70	43	109	60	738	5.7		
**CCU**	40	1	1	6	29	97	103	822	6.4		
**Total**	99	31	14	76	72	206	163	1560	12.1		

In *E. coli* K12 protein-coding genes, the sequences CC[C/U]-[NNN] occur 12,874 times (N = A, C, G and U). Here, each NNN codon that is considered by the published database [42] as a rare codon is analyzed. We chose rare codons whose frequency (freq) of occurrence is less than 1.0 in the database [42]. Of the 12,874 occurrences of total CC[C/U]-[NNN] sequences, 12.1% is made up of a rare codon following CC[C/U]. In this 12.1%, 5.7% is represented by CCC-CNN, CCC-UNN, CCC-ANN, and CCC-GNN, while 6.4% is represented by CCU-CNN, CCU-UNN, CCU-ANN, and CCU-GNN.

## 5. Conclusions

We show here that the natural UGG isoacceptor of tRNA^Pro^, possessing the cmo^5^U34 modified wobble base, is frameshift-prone. This frameshifting propensity arises from the ability of the modified wobble base to pair with multiple codons at the 3rd position. In some mRNA sequences, the natural UGG isoacceptor of tRNA^Pro^ is prone to shift into the +1 frame, which is suppressed by the naturally present post-transcriptionally modified m^1^G37 base on the 3ʹ adjacent position to the anticodon. In other mRNA sequences, the natural UGG isoacceptor can shift into the +2 frame, which is suppressed by the ribosome. This work highlights an active role of the ribosome in maintaining the protein synthesis reading frame.
